# 44. Cost Effectiveness and Clinical Outcomes of Long Acting Lipoglycopeptides Used in Transitions of Care for Deep-Seated Infections

**DOI:** 10.1093/ofid/ofab466.246

**Published:** 2021-12-04

**Authors:** Kayla S Antosz, Julie Ann Justo, Majdi N Al-hasan, Benjamin Tabor, Joseph Kohn, Alexander Milgrom, Kevin Lu, P Brandon Bookstaver

**Affiliations:** 1 Prisma Health Midlands, Columbia, South Carolina; 2 University of South Carolina, Columbia, SC; 3 University of South Carolina College of Pharmacy, Columbia, South Carolina

## Abstract

**Background:**

Dalbavancin and oritavancin are long-acting lipoglycopeptides (LaLGPs) FDA-approved for one-time only dosing for skin and skin structure infections. The use of these agents in serious, deep-seated infections requiring protracted antibiotic courses is of increasing interest. The purpose of this study is to evaluate the economic and clinical utility of LaLGPs in patients requiring protracted antibiotic courses who are not ideal candidates for oral transition or outpatient parenteral antibiotic therapy (OPAT).

**Methods:**

This is a retrospective, observational, matched cohort study of adult patients who received a LaLGP. Patients who received a LaLGP were matched 1:1 to those who received standard of care (SOC) therapy by age (+/- 10 years), infection type, microorganism, and socioeconomic factor (e.g. persons who inject drugs, homelessness). Cost effectiveness was evaluated as total healthcare-related costs between groups. Clinical failure was a composite endpoint of mortality, recurrence, or need for extended antibiotics beyond planned course within 90 days of initial infection. Secondary outcomes included hospital length of stay and proportion of patients who left against medical advice (AMA).

**Results:**

A total of 46 patients were included (23 per group). The most frequent indication was endovascular infection and the most common organism methicillin-resistant *Staphylococcus aureus*. The average length of stay was 22.9 days vs. 31.9 days in the LaLGP and SOC cohorts, respectively (p=0.153). The average total healthcare-related cost of care was USD &295,589 in the LaLGP cohort compared to &326,089 in the SOC cohort (p=0.282). LaLGPs were associated with a mean savings of &30,500 - &55,831 per patient (cumulative cost savings of &701,510). There was no difference in clinical failure between the two cohorts (22% vs. 30%; p=0.491). Nearly 26% of patients in the SOC cohort left AMA compared to 0% in the LaLGP cohort (p=0.022).

**Conclusion:**

Receipt of LaLGPs may be a beneficial treatment option for patients with socioeconomic factors and deep-seated infections who are not candidates for oral transition or OPAT.

**Disclosures:**

**Julie Ann Justo, PharmD, MS, BCPS-AQ ID**, **bioMerieux** (Speaker’s Bureau)**Merck & Co.** (Advisor or Review Panel member)**Therapeutic Research Center** (Speaker’s Bureau)**Vaxart** (Shareholder) **P. Brandon Bookstaver, Pharm D**, **ALK Abello, Inc.** (Grant/Research Support, Advisor or Review Panel member)**Biomerieux** (Speaker’s Bureau)**Kedrion Biopharma** (Grant/Research Support, Advisor or Review Panel member)

